# Development of HPLC Protocol and Simultaneous Quantification of Four Free Flavonoids from *Dracocephalum heterophyllum* Benth.

**DOI:** 10.1155/2015/503139

**Published:** 2015-04-29

**Authors:** Sodik Rakhmonovich Numonov, Muhammad Nasimullah Qureshi, Haji Akber Aisa

**Affiliations:** ^1^Key Laboratory of Plant Resources and Chemistry in Arid Regions, Xinjiang Technical Institute of Physics and Chemistry, Chinese Academy of Sciences, Urumqi 830011, China; ^2^Key Laboratory of Xinjiang Indigenous Medicinal Plants Resource Utilization, Xinjiang Technical Institute of Physics and Chemistry, Chinese Academy of Sciences, Urumqi 830011, China; ^3^State Scientifically-Experimental and Production Organization, Academy of Sciences of the Republic of Tajikistan, 734063 Dushanbe, Tajikistan; ^4^Department of Chemistry, Abdul Wali Khan University, Mardan 23200, Pakistan

## Abstract

Quantification of the four flavonoids, namely, luteolin, kaempferol, diosmetin, and chrysosplenetin, has been performed for the first time in 80% ethanolic extract of *Dracocephalum heterophyllum* B. through HPLC coupled to UV detector after optimization of extracting solvent and chromatographic conditions. Total flavonoids quantified were 0.324 mg/mL of the extract. HPLC analysis delivered contents of the luteolin, kaempferol, diosmetin, and chrysosplenetin as 0.08%, 0.14%, 0.28%, and 0.79% of the dried extract, respectively. LOD (%) values calculated were 0.04, 0.03, 0.03, and 0.08 and LOQ (%) values were 0.08, 0.12, 0.11, and 0.28 for luteolin, kaempferol, diosmetin, and chrysosplenetin, respectively. The recovery percentages for these flavonoids were within the acceptable range of 95% to 105%. Standard deviation and %RSD were calculated for each target analytes individually in extract for determining the reproducibility and accuracy of the method. In no case the %RSD was higher than 1 taking retention time as a factor while in the case of area under the curve maximum %RSD was noted in the case of diosmetin as 2.85. From our literature review regarding the plant species under study, it appears that these flavonoids have not been quantified before and are reported for the first time in this paper.

## 1. Introduction

Medicinal plants have been employed in various traditional medicines throughout the world since ancient time. They have been a rich source of chemicals and thus many bioactive compounds have been isolated in their pure form [1]. Flavonoids are a class of naturally occurring plant secondary metabolites imparting protection to the reservoir [1, 2]. They are compounds of low molecular weight and are chemically polyphenolic in nature presenting a common benzo-*γ*-pyrone structure [3]. They have enormous biological and pharmacological activities conferring many health benefits to the human [1, 2]. They are the group of compounds which received considerable attention from the researchers as depicted from the scientific literature. Mostly they are present in plants as glycosides but can also be isolated in free aglycon form [4, 5].

*Dracocephalum heterophyllum* Benth. is a small perennial aromatic herb belonging to the family Lamiaceae and has been of medicinal importance in Chinese traditional medicine. It is used in traditional way of treatment of tracheitis and cardiovascular disease in Xinjiang and in Tibet region of China [4–6]. The habitat pertains to open and moist slopes. The plant used has been extensively used in Amchi system of medicine in the Ladakh region of the Himalaya for a long period. The decoction of dried flowers and leaves is used in cold, cough, and headache treatment [7]. The essential oil of the plants has been shown to possess antimicrobial and antioxidant activities and thus can be used in cosmetics, food, and pharmaceutical industries [8]. Considering the importance of the phytochemicals responsible for the medical properties of* Dracocephalum heterophyllum* Benth., the investigation was carried out for antioxidative, antidiabetic, and anticancer activity and the presence of phytochemicals with biological activity. Aerial part of* Dracocephalum heterophyllum* is said to contain as many as 10 types of flavonoids, and among these are luteolin, kaempferol, diosmetin, and chrysosplenetin. We have isolated 9 flavonoids from plant which were named chrysosplenetin, diosmetin, luteolin, acacetin 7-*O*-rutinoside, diosmetin 7-*O*-glucoside, rutinoside, kaempferol, kaempferol 3-*O*-*β*-D-glucoside, and quercetin [4, 5]. Various methods have been employed for the determination of flavonoids based on the electrophoresis and chromatography [1, 2, 9–15]. HPLC is the method of choice among the chromatographic techniques for the analysis of flavonoids which needs no derivatization and thus reduces the time consumption in comparison to GC [1, 2, 10]. Moreover, it is safe for flavonoids as it can be operated even at room temperature thus avoiding the risk of decomposition of compounds like flavonoids at high temperature. The present paper deals with the quantification of four flavonoids, namely, luteolin, kaempferol, diosmetin, and chrysosplenetin, which are available in free aglycon forms in the plant under study, using the HPLC in a single run. Further, this is the first paper describing the quantification of these four flavonoids in the plant extract under study.

## 2. Experimental

### 2.1. Chemicals and Reagents

The aerial parts of* Dracocephalum heterophyllum* were collected from Atush in Xinjiang, China, in August 2010, respectively. After being air-dried in nature, the plant was ground with a laboratory mill and then passed through a 20-mesh sieve. The standards of chrysosplenetin, diosmetin, kaempferol, and luteolin were isolated from* D. heterophyllum* in the pure form. Acetonitrile, HPLC grade, was purchased from LiChrosolv, Merck, Darmstadt, Germany; the methanol and formic acid (LiChrosolv, Merck, Darmstadt, Germany) were of analytical reagent grade.

### 2.2. Extraction

Extraction was performed according to the already published procedure in the literature [4]. Extraction efficiency was determined based on the total extractive percent value using different compositions of aqueous ethanol as 50%, 70%, 80%, and 95% at room temperature for 48 hours. Afterwards, one hundred grams of above ground part of* D. heterophyllum* was weighed accurately and extracted with 400 mL of 80% aqueous ethanol for 48 hours at room temperature. The extract was filtered, decolorized, and defatted by petroleum ether for several times. The extract was reduced to dryness through rotary evaporator under reduced pressure yielding 20 mg dried extract. The extract was reconstituted in 1 mL of pure methanol and injected 10 *μ*L into the column.

### 2.3. Determination of Total Flavonoids

Total flavonoids contents were determined using the method of Chang et al. 2002 [16]. One g of plant material was extracted with 50 mL of methanol under reflux and the extract obtained was reduced to dryness. The residue was reconstituted in 10 mL of methanol and this extract was used for the determination of total flavonoids contents. Briefly, 0.5 mL of plant extract/standard solution was mixed with 1.5 mL of methanol in a test tube. 0.1 mL of 10% aluminum chloride, 0.1 mL of 1 M potassium acetate, and 2.8 mL of distilled water were added to the test tube and mixed thoroughly after each addition. It was allowed to react for flavonoid-aluminum complex formation for 30 minutes at room temperature. Methanol was used as blank and preceded in the same way as above. Afterwards, the absorbances of the reaction mixtures were measured at 415 nm using the UV-visible spectrophotometer. Quercetin was used as standard and six working standard solutions were prepared in the concentration range 0.01 mg/mL to 0.1 mg/mL for constructing the calibration curve.

### 2.4. Preparation of Standard Solutions

The four flavonoid standards, that is, luteolin, kaempferol, diosmetin, and chrysosplenetin, were mixed and stock solution of standard flavonoids mixture was prepared in methanol having 1 mg/mL concentration of each standard. From this stock solution, four working standard solutions with a concentration range 0.0001–1 mg/mL were prepared.

### 2.5. Instrumentation

HPLC analysis was performed using a DIONEX Ultimate 3000 HPLC system (Thermo-Fisher, USA) equipped with autosampler and coupled to variable UV wavelength detector. The chromatography was performed on Sunfire C18 column from Waters, USA, having the following specifications: internal diameter 4.6 mm, height 250 mm, and particle size 5 *μ*m. The chromatographic column was protected by a Sunfire C18 guard column of the following dimensions: internal diameter 4.6 mm, height 20 mm, and particle size 5 *μ*m obtained from Waters, USA. The control of the instrument and the data analysis was performed by Chromeleon version 7.2 software provided by the supplier.

### 2.6. Optimization of the Conditions and Chromatographic Separation of the Flavonoids

Chromatographic conditions were optimized in order to reach baseline separated peaks of the target analytes. For this purpose, different mobile phases with varying gradient elution were employed. The mobile phase consisting of A: methanol and B: 0.1% (*v*/*v*) formic acid in water with the gradient set up as mentioned below delivered good baseline separation of the targeted peaks. The gradient used was as follows: zero time condition was 8% A and it was increased to 78% A in 50 minutes. The column conditioning and equilibration were performed in 5 minutes attaining the initial conditions. 10 *μ*L of the sample extract and the standards were injected into the chromatographic column maintained at 35°C. The flow rate used was 1 mL/min and detection of the eluted peaks was performed at 254 nm. The analyses were performed five times.

## 3. Results and Discussion

### 3.1. Optimization of the Extraction Procedure

**Figure 1:**
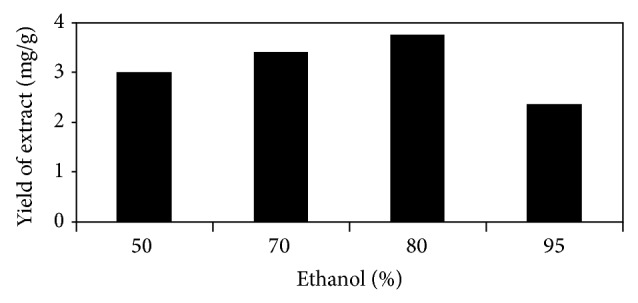
Comparison of the extraction efficiencies of various compositions of aqueous ethanol.

The method of extraction by soaking the plant material in solvent was selected due to its simplicity and easy manageability. Ethanol in combination with water was used as extracting solvent because of its less toxicity and easy availability. Different combinations of ethanol with water as 50%, 70%, 80%, and 95% aqueous ethanol were evaluated in order to determine the extraction efficiency and to reach optimized solvent for extraction.  Figure 1 shows the graphical comparison of the extraction yields of various compositions of aqueous ethanol. 80% ethanol delivered the highest extraction yield (3.76 mg/g) among the tested solvents. The extractive values for 50%, 70%, and 95% ethanol were 3 mg/g, 3.4 mg/g, and 2.35 mg/g, respectively, from dry* D. heterophyllum* plant.

### 3.2. Determination of Total Flavonoids

Total flavonoids contents were determined using the published method in the literature using AlCl_3_ as complexation reagent forming a complex with flavonoids which has a maximum absorption at 415 nm [16]. Total flavonoids contents were quantified as equivalent of quercetin and calibration curve was produced with an *R* ^2^ value 0.9971. The calibration curve was passed through zero and the contents of flavonoids were calculated using the regression equation obtained from the calibration curve. The total flavonoids contents yielded in the aerial part of* D. heterophyllum* were 0.324 mg/mL of the final extract.

### 3.3. Optimization of the Chromatographic Conditions

**Figure 2:**
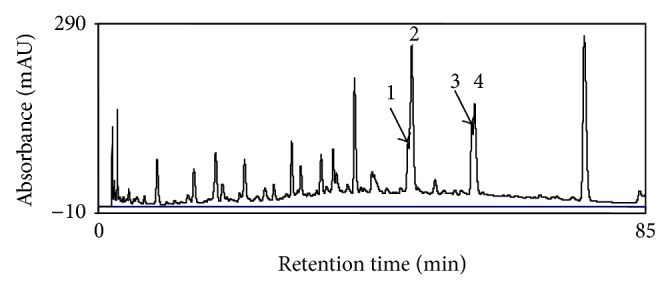
HPLC chromatogram of 80% ethanol extract of* D. heterophyllum*. Mobile phase: acetonitrile, 0.1% HCOOH in water; flow rate: 1 mL/min; detection: 254 nm. 1: luteolin, 2: kaempferol, 3: diosmetin, and 4: chrysosplenetin.

Preliminary separation of the standard mixture and the extract was performed with mobile phase consisting acetonitrile and 0.1% (*v*/*v*) formic acid in water using the gradient mode of elution. This mobile phase did not deliver good resolution as in Figure 2 it is clear that at 48 min two target peaks of luteolin and quercetin and at 58 min two target peaks of diosmetin and kaempferol are coeluting and are merged. Therefore, quantification of these four flavonoids was not possible as baseline separated peaks are required for the quantitative determination.

**Figure 3:**
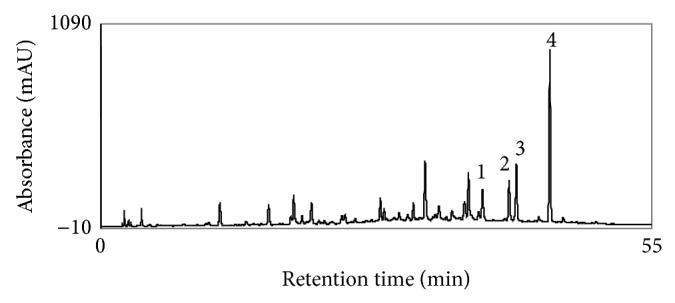
HPLC chromatogram of 80% ethanol extract of* D. heterophyllum.* Mobile phase: methanol, 0.1% HCOOH in water; flow rate: 1 mL/min; detection: 254 nm. 1: Luteolin, 2: kaempferol, 3: diosmetin, and 4: chrysosplenetin.

Therefore, the mobile phase was changed to methanol and 0.1% (*v*/*v*) formic acid in water using the conditions as described in experimental section. With this optimum baseline separation of the target analytes was achieved enabling the quantification in the sample extract (Figure 3). The peaks of target flavonoids luteolin, kaempferol, diosmetin, and chrysosplenetin eluted at retention times 38.8 min, 41.5 min, 42.3 min, and 45.8 min, respectively, in the sample extract.

### 3.4. Quantification of Flavonoids

Using the optimized chromatographic conditions calibration curves for the four flavonoids were established through analyzing working standard solutions in triplicate. Regression equations and *R* ^2^ values and percentage concentrations of the flavonoids in the plant extract obtained from these analyses are tabulated in Table 1. Contents of individual flavonoids were calculated as percentage of the dried extract using the regression equations.

Contents of the diosmetin (0.28%) and chrysosplenetin (0.79%) were significant and luteolin yielded the lowest amount (0.08%) in this study while the amount of kaempferol was 0.14%. Standard deviation and the %RSD are the results of the five replicate injections of the plant extracts. LOD and LOQ were calculated using Microsoft Excel sheet based on standard deviation (Table 1). Reproducibility and accuracy of the method were tested by analyzing the sample extract five times and their standard deviation and %RSD were calculated based on retention times and area under the curve for each target analytes individually.  Table 2 shows the data obtained from the five replicate injections of the sample extract. In no case, the %RSD was higher than 1 taking retention time as a factor while in the case of area under the curve maximum %RSD was noted in the case of diosmetin as 2.85 (Table 2). Percent recovery of the optimized HPLC method was determined through injecting the two concentrations (1 mg/mL and 0.1 mg/mL) of each individual flavonoid standard under study. This experiment resulted in the recovery percentage within the acceptable range of 95% to 105% according to the ICH guidelines for validation of analytical methods [17]. From our literature review regarding the plant species under study, it appears that these flavonoids have not been quantified before and are reported for the first time in this paper.

## 4. Conclusions

The study proved the presence of biologically and pharmacologically important flavonoids in quantifiable amount making the plant* Dracocephalum heterophyllum *B. beneficial for the preparation of phytopharmacon. The results of the study can be used for developing the quality control profile by the pharmaceutical and phytopharmaceutical industries. Further, the method applied is reliable and reproducible and can be used for the determination of flavonoids in plant extracts.

## Figures and Tables

**Table 1:** Results of the quantification of flavonoids in *Dracocephalum  heterophyllum* Benth.

Flavonoid	Regression equation	*R* ^2^ value	Percentage^∗∗^	SD^∗^	RSD^∗^	LOD^∗^ (%)	LOQ^∗^ (%)
Luteolin	*y* = 298.53*x* + 30.661	0.9998	0.08	0.01	0.15	0.04	0.08
Kaempferol	*y* = 335.3*x* + 29.891	0.9999	0.14	0.01	0.08	0.03	0.12
Diosmetin	*y* = 788.3*x* + 14.623	0.9999	0.28	0.01	0.04	0.03	0.11
Chrysosplenetin	*y* = 741.7*x* + 47.374	0.9998	0.79	0.03	0.04	0.08	0.28

^∗^SD: standard deviation; RSD: relative standard deviation; LOD: limit of detection; LOQ: limit of quantification; ^∗∗^percentage of the dried extract.

**Table 2:** Data obtained from the five replicate HPLC analyses for accuracy and reproducibility.

Number of replicates	Luteolin		Kaempferol		Diosmetin		Chrysosplenetin	
	Rt^∗^	Area	Rt^∗^	Area	Rt^∗^	Area	Rt^∗^	Area
1	38.85	34.58	41.58	38.90	42.34	56.63	45.78	158.62
2	38.88	35.08	41.62	39.48	42.37	57.93	45.80	162.15
3	38.85	35.64	41.58	40.02	42.34	59.09	45.80	165.60
4	38.87	36.29	41.60	40.62	42.35	60.36	45.78	169.33
5	38.90	36.20	41.64	38.75	42.38	56.46	45.83	166.19

Total	194.35	177.79	208.02	197.77	211.78	290.47	228.99	821.89
Average	38.87	35.55	41.60	39.55	42.35	58.09	45.79	164.37
SD	0.02	0.73	0.02	0.77	0.01	1.65	0.02	4.10
RSD%	0.05	2.05	0.06	1.97	0.04	2.85	0.04	2.49

^∗^Rt: retention time.
